# Salud bucal en la primera infancia: estrategia con agentes educativas y acudientes*

**DOI:** 10.15649/cuidarte.2676

**Published:** 2023-09-06

**Authors:** Alexandra Agudelo Ramírez, Johnny Alexander Galvis Aricapa, Edwin Villegas García

**Affiliations:** 1 . Grupo de investigación GISCO, Institución Universitaria Visión de las Américas. Pereira, Colombia. Email: alexandra.agudelo@uam.edu.co Institución Universitaria Visión de las Américas Pereira Colombia alexandra.agudelo@uam.edu.co; 2 . Grupo de investigación GISCO, Institución Universitaria Visión de las Américas. Pereira, Colombia. E-mail: johnny.galvis@uam.edu.co Institución Universitaria Visión de las Américas Pereira Colombia johnny.galvis@uam.edu.co; 3 . Grupo de investigación GISCO, Institución Universitaria Visión de las Américas. Pereira, Colombia. Email: edwin.villegas@uam.edu.co Institución Universitaria Visión de las Américas Pereira Colombia edwin.villegas@uam.edu.co

**Keywords:** Educación para la Salud, Salud Bucal, Promoción de la Salud, Investigación Participativa Basada en la Comunidad, Primera Infancia., Health Education, Oral health, Health Promotion, Community Based Participatory Research, Early Childhood, Health Education, Oral health, Health Promotion, Community Based Participatory Research, Early Childhood

## Abstract

**Introducción:**

La dentición temporal es esencial para la nutrición, habla y autoestima. Los niños y niñas menores de 5 años no poseen habilidades motoras para efectuar higiene bucal por sí mismos.

**Objetivo:**

Diseñar e implementar una estrategia educativa para la promoción de la salud bucal en la primera infancia con agentes educativas y acudientes de los niños y niñas que asisten a los hogares comunitarios del Instituto Colombiano de Bienestar Familiar en Santa Rosa de Cabal (Colombia) en el 2019.

**Materiales y métodos:**

Investigación cualitativa, diseño investigación-acción. Muestreo no probabilístico por conveniencia, participaron 25 agentes educativas y 55 acudientes. Técnicas de investigación empleadas: grupos focales y talleres pedagógicos. Para analizar los datos se usó Análisis Temático.

**Resultados:**

Se desarrolló en 3 fases. 1) los/as participantes expresaron que tener salud bucal consiste en la adecuada higiene, aunque todos/as conocían la caries, los cuidadores no la comprendían como una patología, las agentes educativas percibieron tener alta capacidad de autoeficacia para contribuir a la salud bucal de los niños/as. 2) En los talleres pedagógicos se resolvieron inquietudes en temas como crecimiento y desarrollo del sistema estomatognático, enfermedades bucales, entre otros. 3) Las agentes educativas aprendieron la relación de la salud bucal con lactancia materna, asimismo las causas y consecuencias de las patologías bucales, y replicaron lo aprendido.

**Conclusiones:**

Se recomienda el desarrollo de estrategias educativas guiadas por modelos teóricos y con metodologías participativas, adaptadas a las necesidades y contextos de las comunidades, pues se requiere producir conocimiento situado y con aceptabilidad cultural.

## Abstract (en)

**Introduction:**

Primary dentition is essential for nutrition, speech, and self-esteem. Children under the age of 5 do not have the motor skills to perform oral hygiene on their own.

**Objective:**

To design and implement an educational strategy to promote oral health in early childhood with educators and parents/guardians of children attending the community homes of the Colombian Family Welfare Institute in Santa Rosa de Cabal (Colombia) in 2019.

**Materials and methods:**

Qualitative, action research design. Non-probability convenience sampling was used. Twenty-five educators and 55 parents/guardians participated. Focus groups and pedagogical workshops were the research techniques used. Thematic analysis was performed for data analysis.

**Results:**

It was conducted in three phases: 1) Participants expressed that oral health is about proper hygiene. Although all were aware of caries, the caregivers did not understand it as a pathology. The educators perceived that they had high self-efficacy to contribute to children's oral health; 2) in the pedagogical workshops, concerns were resolved on topics such as growth and development of the stomatognathic system, oral diseases, among others, and 3) the educators learned about the relationship between oral health and breastfeeding, as well as the causes and consequences of oral pathologies, and reproduced what they learned.

**Conclusions:**

The development of educational strategies based on theoretical models and participatory methods adapted to the needs and contexts of the communities is recommended since it is necessary to produce situated knowledge with cultural acceptance.

## Resumo

**Introdujo:**

A dentigáo temporaria é essencial para nutrigáo, fala e autoestima. Meninos e meninas menores de 5 anos náo possuem habilidades motoras para realizar a higiene bucal sozinhos.

**Objetivo:**

Desenhar e implementar uma estratégia educativa para a promogáo da saúde bucal na primeira infancia com agentes educativos e responsáveis dos meninos e meninas que frequentam as casas comunitárias do Instituto Colombiano de Bem-Estar Familiar em Santa Rosa de Cabal (Colombia) em 2019.

**Materiais e Métodos:**

Pesquisa qualitativa, desenho de pesquisa-agáo. Amostragem náo probabilística por conveniencia, participaram 25 agentes educativos e 55 responsáveis. Técnicas de pesquisa utilizadas: grupos focais e seminários educativos. Para analisar os dados se usou Análises Temático.

**Resultados:**

Foi desenvolvido em 3 fases. 1) os participantes expressaram que ter saúde bucal consiste em higiene adequada, embora todos soubessem sobre a cárie, os cuidadores náo a entendiam como uma patologia, os agentes educativos percebiam que tinham alta capacidade de autoeficácia para contribuir com a saúde bucal das criangas 2) Nos seminários educativos foram sanadas dúvidas sobre temas como crescimento e desenvolvimento do sistema estomatognático, doengas bucais, entre outros. 3) Os agentes educativos aprenderam a relagáo entre saúde oral e amamentagáo, bem como as causas e consequencias das patologias orais, e replicaram o que aprenderam.

**Conclusóes:**

Recomenda-se o desenvolvimento de estratégias educativas pautadas em modelos teóricos e com metodologias participativas, adaptadas as necessidades e contextos das comunidades, pois é necessário produzir conhecimento situado com aceitabilidade cultural.

## Introducción

Los niños y niñas con edades entre 0 y 5 años poseen dentición temporal, la cual requiere de cuidados para garantizar apropiada nutrición, masticación, habla, apariencia y autoestima^1^. Las alteraciones de la salud bucal a temprana edad impactan la calidad de vida de los infantes y sus familias, los sistemas de salud y las sociedades.

La caries en dientes deciduos es la enfermedad número 12 más prevalente del mundo y afecta a 560 millones de niños y niñas^2^, su prevalencia entre los 3 y 5 años varía entre países; por ejemplo, mientras que en Reino Unido la caries visible es del 12 %, en Camboya e Indonesia es del 90 %^3^. También, puede causar ausentismo escolar y laboral, y se asocia con la desnutrición de los infantes en países de bajos y medianos ingresos^4^.

La prevalencia de caries en América se calculó entre 40 % y 85 % en el 2017. En Colombia la prevalencia modificada de caries en menores entre 1 y 5 años según el Estudio Nacional de Salud Bucal IV fue de 61,92 %^5^. Entre tanto, en el municipio de Santa Rosa de Cabal (Colombia), lugar donde se desarrolló el presente proyecto, se realizó una investigación en hogares comunitarios del Instituto Colombiano de Bienestar Familiar (ICBF) la cual reflejó que la prevalencia modificada de caries en niños/as menores de 6 años fue del 63,6 %^6^.

Cabe mencionar que el papel de los cuidadores en la prevención de enfermedades bucales en los infantes es crucial^1^. En Colombia se han hecho estudios con agentes educativas de ICBF y acudientes en los que se reportan falta de información sobre estrategias para el cuidado de la salud bucal^7^, aunque se reconocen saberes sobre algunas patologías bucales en la primera infancia^6^^,^^8^. Igualmente, se manifiesta mejoría en aquellos niños/as atendidos por cuidadores con mayor conocimiento del tema^9^.

La caries es un problema de salud pública, ya que es prevenible con intervenciones costo efectivas; en tanto, los tratamientos no son asequibles para amplios sectores de la población^4^. Al respecto, una revisión sistemática de intervenciones para el correcto cepillado dental en menores de 6 años demostró que aquellas con enfoque pedagógico fueron las más eficaces^10^. También, fue demostrado que la capacitación a maestros y padres/madres de familia es una estrategia efectiva para la higiene bucal^11^^,^^12^. Sin embargo, son pocos los estudios con metodologías participativas, entre los que se destacan trabajos con Investigación Participativa Basada en Comunidad^13^^,^^14^ y otros con relevancia cultural y comunitaria^15^^-^^17^.

Por otro lado, se tomó como referente conceptual el Modelo de Creencias en Salud (MCS), el cual explica las conductas de los individuos para prevenir la enfermedad o buscar el restablecimiento de su bienestar^18^. La decisión de adoptar estos comportamientos de manera permanente depende de las percepciones sobre la susceptibilidad y severidad de la patología, y de los beneficios y barreras para realizar hábitos saludables^19^. En 1988 se agregó el componente de autoeficacia, definida como la creencia de ser capaz de ejecutar exitosamente un comportamiento recomendado^20^. Diferentes autores sugieren este modelo en proyectos de promoción de la salud^21^^,^^22^.

En consecuencia, el objetivo de la investigación fue diseñar e implementar una intervención educativa para la promoción de la salud bucal de la primera infancia con las agentes educativas y acudientes de los niños y niñas que asisten a los hogares comunitarios del ICBF en Santa Rosa de Cabal (Colombia) en el 2019.

## Materiales y Métodos

El estudio tuvo enfoque cualitativo y se utilizó el método Investigación Acción (IA). El método se compone de fases cíclicas de planeación, acción, observación y reflexión, por lo que es flexible y se ajusta a los problemas y procesos específicos de las comunidades^23^.

La población fueron las agentes educativas del ICBF en el municipio de Santa Rosa de Cabal, quienes trabajan en 14 hogares comunitarios y dos Centros de Desarrollo Infantil. Fueron elegidas dada la histórica relación interinstitucional entre ICBF y la universidad a la cual pertenecen los autores de este artículo. Participaron 25 agentes educativas quienes seleccionaron a 55 acudientes que requerían capacitación. Todos los involucrados firmaron consentimiento informado.

El procedimiento se desarrolló en etapas:

*Fase 1, reconocimiento de saberes y experiencias: se conformaron grupos focales para conocer en profundidad las experiencias, percepciones y conocimientos acerca de la salud bucal en la primera infancia. La información se usó como insumo para la etapa siguiente.*
*Fase 2, desarrollo de la intervención educativa: se diseñó e implementó una intervención educativa con dos componentes, uno con las agentes educativas y otro con los acudientes.*
*Fase 3, indagación de aprendizajes y cambios: para evidenciar los cambios en prácticas y percepciones, además de los aprendizajes y aceptabilidad de la intervención, se realizaron grupos focales con las agentes educativas.*

Las técnicas de investigación utilizadas fueron:

Grupos focales: se desarrollaron cuatro con agentes educativas, dos en la fase 1 y dos en la fase 3. En la primera fase se efectuaron dos con acudientes. En cada uno participaron aproximadamente 12 personas. Las preguntas se formularon de acuerdo con el MCS y fueron adaptadas al lenguaje de los participantes.

Talleres pedagógicos: se usaron en la fase educativa, la información se recolectó en diarios de campo.

Los datos fueron transcritos, la información obtenida con ambas técnicas tuvo el mismo tratamiento y para su análisis no se usó software especializado. Los datos se encuentran almacenados en un repositorio público^24^. Se procedió con la identificación de las unidades de sentido, codificación y categorización a través del método de Análisis Temático^25^. El proceso analítico inició con la individualización de las unidades de sentido, las cuales fueron comparadas constantemente. Esto dio paso a la creación de códigos, es decir, a las etiquetas para organizar la información. Estos a su vez se relacionaron con el referente teórico para la clasificación de la información en categorías, de las cuales se derivó el proceso interpretativo.

El análisis fue realizado por la investigadora principal y revisado por los otros dos investigadores. No fue posible realizar la devolución sistemática de los resultados dado que a inicios del 2020 se retrasó la contratación de ICBF y comenzó la pandemia por COVID-19.

La investigación se clasificó con riesgo mínimo según la resolución 8430 de 1993 del Ministerio de Salud de Colombia. Además, recibió aval del Comité de Ética en Investigación de la Institución Universitaria Visión de las Américas y fue avalada por las directivas de ICBF en Santa Rosa de Cabal.

## Resultados

Se presentan según las fases metodológicas desarrolladas en un periodo de siete meses. Los testimonios de las/os participantes se exponen en tablas. En la Tabla 1 se muestran los códigos y categorías utilizadas en el análisis de datos.

**Tabla 1:** Matriz de categorías y códigos y su relación con las preguntas orientadoras de los grupos focales.

Categorías	Códigos	Preguntas de los grupos focales (adaptadas a agentes educativas y acudientes)
Creencias en salud bucal (generalidades sobre el Modelo de Creencias en Salud)	Creencias sobre la salud bucal y, en particular, la de los niños y niñas	¿Qué es una buena salud bucal para usted? ¿Cuál es la diferencia entre la salud bucal de un adulto comparada con la de los niños y niñas?
		¿Cuáles son las causas de la buena o la mala salud bucal en la primera infancia?
		¿Qué sugerencias tiene para la fase educativa a desarrollarse?
	Opiniones del proceso educativo	Posterior a la fase educativa: ¿Cuáles son sus opiniones sobre el proceso educativo que se desarrolló en este proyecto? ¿Qué resultados ve usted después de este proceso educativo?
Susceptibilidad percibida	¿Le puede dar a los niños/as de su hogar comunitario (su hijo/a) alguna enfermedad/ lesión en la cavidad bucal?	¿Cree que los niños y niñas de su hogar comunitario (su hijo/a) tienen alguna enfermedad en la boca? ¿Cree que el bienestar de los niños y niñas se puede ver afectado por algún problema en la boca?
Severidad percibida	¿Por qué son graves esas enfermedades? ¿Qué consecuencias tienen?	¿Qué opina de la caries en los niños y niñas? ¿Qué consecuencias trae?
		¿Qué otros problemas pueden tener los niños y niñas en la boca? ¿Qué consecuencias traen?
Beneficios percibidos	¿Qué beneficios tienen los niños y niñas si hay cuidado bucal? ¿Qué beneficios tienen las agentes educativa y acudientes?	¿Qué beneficios le trae a los niños y niñas tener la boca sana? ¿Qué beneficios le trae a usted (agente educativa/acudiente) que los niños y niñas tengan la boca sana?
Barreras percibidas	¿Qué barreras tienen las agentes educativas y los acudientes para el cuidado bucal de los infantes?	¿Qué dificultades tiene para cuidar la salud bucal de los niños y niñas de su hogar comunitario (de su hijo/a)?
Autoeficacia	¿Cree que puede realizar la conducta? ¿Sirve realizarla? (eficacia percibida de la conducta)	¿Quién cree que es responsable de cuidar la salud bucal de los niños y niñas?
	¿Tiene la habilidad para realizar la conducta?	¿Siente que puede ayudar mantener la salud bucal de los niños y niñas de su hogar comunitario (su hijo/a)? ¿ Usted como agente educativa/acudiente de qué manera puede prevenir problemas de salud bucal de los niños y niñas?
		¿Cómo han sido sus experiencias acompañando a los niños y niñas en el cepillado y enseñándoles a cepillarse solo/a? Lo mismo con la seda dental.

Fase 1:

Resultados de los grupos focales con agentes educativas (ver Tabla 2).

**Tabla 2:** Fase 1, unidades de sentido identificadas en los grupos focales con agentes educativas.

Categorías	Unidades de sentido
Creencias en Salud Bucal	“Anteriormente los padres no cuidaban los dientecitos de leche que porque eso se caía (...) desde ahora los niños tienen los dientecitos y es necesario tratarlos bien, porque si no esos dientecitos de leche perjudican los otros”. (AE12) “Nosotras no conocemos las enfermedades más allá al hablar de la caries y entonces si nosotras tuviéramos ese conocimiento de enfermedades, de consecuencias, podríamos hablarles con más claridad a los acudientes”. (AE11) “¿Qué tan cierto es que los dientes de leche totalmente podridos pueden dañar los de hueso? y ¿qué tantas consecuencias pueden traer?”. (AE8) “¿El tetero es malo para la nutrición?”. (AE18) “¿Qué es de cierto que cuando el niño le retiran los dientes de leche antes de tiempo se le demora más los dientes permanentes en salir?”. (AE9)
Susceptibilidad Percibida	“La caries, sé que es como acumulación de alimentos que empieza a podrir el diente, se come todos los dientes, en la noche actúa con más rapidez y es súper peligrosa, yo le he leído que pueden llegar hasta el cerebro las enfermedades bucales y qué susto”. (AE21)
Severidad Percibida	“Hemos tenido casos de niños que han tenido los dientes totalmente dañados, entonces entre ellos mismos el niño habla y los otros niños: "ay vea tiene los dientes sucios". Le va a dar pena hablar, le va a dar pena reírse”. (AE4)
Barreras Percibidas	“Madres que ni saben cepillar los niños de 2, de 3 años, y nos ha pasado a muchas que el niño llega al hogar comunitario y uno le dice “¿amor, se cepilló los dientes?” y “no, mi mamá no me deja cepillar; no, mi mamá no me da crema””. (AE2)
Beneficios Percibidos	"Si ellos están bien entonces no les va a dar pena mostrar los dientecitos al reír". (A5)
Autoeficacia Percibida	“A los niños les encanta cepillarse y, es más, a uno le da pesar porque hay niños que ni siquiera saben coger el cepillo. Y hay niños que por ejemplo les va uno a cepillar la lengüita y hasta les da náuseas porque nunca les han cepillado la lengua (.)”. (AE6) “El cepillado en el hogar [comunitario] se convierte hasta en un juego, dependiendo cómo nosotras lo realicemos. Por ejemplo, yo les tengo una canción y ellos ya saben cuándo nos vamos a cepillar”. (AE10) “Las profes son las que identifican que de pronto tienen los dientecitos malitos y empiezan hacer el acercamiento con los papitos para decirle que hay que pedir cita médica, llevarlos a odontología y también cuando llega un niño por primera vez se le pide un vaso, el cepillo y la toallita para poder empezar a hacer las prácticas y hay papás muy juiciosos, pero hay otros que no son tanto”. (AE16)

### Creencias en Salud Bucal

Las agentes coincidieron en que tener buena salud es cepillarse tres veces al día, usar seda dental, cambiar con frecuencia el cepillo y asistir regularmente al odontólogo. Algunas la definieron como tener los dientes sanos y la ausencia de enfermedades. Manifestaron la importancia de asistir a la consulta odontológica con el bebé desde su nacimiento o desde que saliera el primer diente, enfatizaron en el cuidado de los dientes temporales e hicieron hincapié en la necesidad de involucrar a los acudientes en este proyecto.

También, expresaron inquietudes acerca de implementos de limpieza, crecimiento y desarrollo, patologías diferentes a la caries, hábitos parafuncionales, nutrición e ingesta de medicamentos. Todas estas dudas se resolvieron en los talleres pedagógicos.

### Susceptibilidad Percibida

Todas las agentes conocen la caries y han tenido niños/as en sus hogares con esta enfermedad. Son conscientes del riesgo de la patología, pues la asocian con pérdidas dentales, dolor e infección. Igualmente, mencionaron que es común que a los infantes les den "sapos" o "llagas", sangrado en las encías, amigdalitis, herpes o problemas relacionados con hábitos como el chupo o el tetero. Además, expresaron desconocer por qué se presentan algunas enfermedades y sus consecuencias.

### Severidad Percibida

Las agentes afirmaron que la caries ocasiona traumas en los niños/as debido al fuerte dolor y al tener que asistir al odontólogo. Por lo anterior, muchos de los infantes han desarrollado miedo a la consulta odontológica. Manifestaron que algunos padres/madres deciden no llevar a sus hijos/as al centro de salud, lo que genera aún más complicaciones.

Otras consecuencias de la caries que las agentes identificaron fueron las burlas de los compañeros, lo que afecta la autoestima e interfiere en su proceso de socialización, también las dificultades para masticar y comer con normalidad, la pérdida de piezas dentales y la halitosis.

### Barreras Percibidas

Las agentes afirmaron que, aunque enseñan la práctica del cepillado dental, se hace difícil hacerlo de manera personalizada pues cada una está encargada de un grupo entre 12 y 20 niños/as, esto implica que ninguna use seda dental con ellos. Otro aspecto referido fue la falta de compromiso de los acudientes para adoptar hábitos de higiene bucal, algunos debido a que trabajan la mayor parte del día, otros por dificultades económicas para comprar implementos de higiene, otros por desconocimiento de cómo efectuar la higiene bucal de sus hijos/as y las consecuencias de no hacerlo. Manifestaron que muchos acudientes desconocen la necesidad de asistir a las consultas odontológicas preventivas con los niños/as.

### Beneficios Percibidos

Esta fue una de las dimensiones que poco surgió en el diálogo. Implícitamente se entiende que las agentes educativas consideran que la salud bucal hace parte del bienestar general de los niños/as, y que esto les garantiza la posibilidad de socializar con sus pares, de construir autoestima y autoimagen asertiva. Asimismo, en sus discursos se identifica que tener la boca sana evita episodios de dolor, enfermedad y consultas por urgencias o complicaciones como "dolor de muela".

### Autoeficacia Percibida

Las agentes percibieron que tienen alta capacidad de autoeficacia pues se sienten capaces de contribuir a la salud bucal de los niños/as, sin embargo, creen que para ello requieren la cooperación de los acudientes. Ellas consideran que tienen los conocimientos básicos para acompañar el proceso educativo de los padres/madres, pero desean capacitarse para tener argumentos más persuasivos.

Las experiencias sobre el cepillado en el hogar comunitario son en general positivas, dado que a la mayoría de los niños/as les gusta cepillarse. Para ello algunas agentes emplean canciones y juegos, aunque se enfrentan a dificultades como la ingesta de la crema dental, la mordida al cepillo o el reflejo vomitivo al limpiar la lengua.

A continuación, se muestran los resultados con acudientes (ver Tabla 3).

**Tabla 3:** Fase 1, resultados de los grupos focales con acudientes.

Categorías	Análisis de las categorías	Unidades de sentido
Creencias en Salud Bucal	Para ellos la salud bucal se centra en el cepillado tres veces diarias y citas odontológicas periódicas. Las diferencias que perciben acerca de la salud bucal de los adultos en comparación con los infantes se atribuyeron al tipo de dentición, a que la crema dental que usan los adultos "es más fuerte", que los "dientes de leche" son más delicados y que dependen del cuidado de los acudientes. Al preguntar qué querían aprender sobre la salud bucal en la primera infancia surgieron numerosos interrogantes relacionados con las causas de enfermedades, qué hacer ante manifestaciones bucales, técnicas de cepillado, uso de seda dental, problemas bucales hereditarios y caída de los dientes. Algunas sugirieron que las sesiones fueran prácticas, que incluyeran imágenes y videos para ilustrar los temas.	“Quiero aprender cómo cuidar la boquita de los niños, por ejemplo, si los niños tienen las encías hinchadas no sabemos qué hacer”. (C3) “¿Desde qué edad es importante usar la seda dental en los niños?”. (C18)
Susceptibilidad Percibida	Al preguntarles si creían que sus hijos/as tenían alguna enfermedad en la boca, respondieron al unísono negativamente. Adicionalmente, las "llagas o sapos" (aftas) no las consideraron enfermedad sino situaciones eventuales. Llama la atención que ninguna mencionó que su hijo/a tuviera caries o gingivitis. Las madres también refirieron otro tipo de problemas como uso del tetero, la succión digital y la onicofagia, al tiempo que reconocieron que en el hogar comunitario se realizan esfuerzos por evitar dichos problemas. Varias de las participantes relataron anécdotas de traumas en los que sus hijos/as perdieron piezas dentales o tuvieron muerte pulpar.	“Los sapos, cuando a la niña le daban se le ponía mucho la lengüita amarilla. Ella hace poquitico tuvo una amigdalitis severa y le dolía tanto las amígdalas y le daba tanta fiebre que se le hicieron llaguitas en la encía y no era capaz de comer nada”. (C19) “La mía sí toma tetero, pero ya desde que está en el jardín lo ha ido mermando, *(...)* ese dedo es cosa verraca, toda la noche chupa dedo”. (C3)
Severidad Percibida	A pesar de que reconocen las consecuencias de las caries, no se refieren a ella como una enfermedad y algunas no tienen claro por qué sucede. Conocen que este problema conlleva a pérdida de piezas dentales, infección en los huesos, cambios de color y forma de los dientes que evoluciona a estadios más críticos que afectan incluso al corazón o la calidad de vida. Cuando se les preguntó por otros problemas que los niños/as podrían tener, respondieron "cálculos" y "sapos". Acerca de las consecuencias, mencionaron las afectaciones emocionales que pueden acarrear en los niños/as.	“De pronto cuando estén más grandes se van a sentir mal con los otros niños que tengan los dientes buenos ¿cierto? empiezan a hacerle bullying o algo, se sienten tristes”. (C16)
Barreras Percibidas	Una de las dificultades reiterativas fue la falta de atención humanizada en la consulta odontológica. Afirmaron que sus hijos/ as han sido atendidos con métodos toscos y que les han practicado exodoncias sin anestesia, lo que provoca temores que impiden a los niños/as regresar. Otra de las barreras más comunes es que los niños/as se quedan dormidos en la noche después de consumir el biberón y los acudientes no los despiertan para cepillarlos. Además, mencionaron que algunos niños/as no les gusta cepillarse, es difícil cepillar la lengua, ingieren la crema dental o no agarran adecuadamente el cepillo.	"Ella se duerme, o a veces come y cuando yo voy la miro está por allá durmiendo, entonces ahí sí grave. Ah voy y la cobijo, qué pesar (risas), yo no la voy a levantar para lavarle los dientes". (C23) "Le sigue fastidiando una muelita, sino que cuando la llevo a odontología a ella no le ponen anestesia, sino que comienzan a rasparle así sin eso y a ella le da mucho miedo". (C13)
Autoeficacia	Coincidieron en que los principales responsables de cuidar la salud "Él tiene tres años no tiene caries ni nada bucal de los niños/as son los acudientes. Sin embargo, sienten que y yo creo pues que estoy haciendo bien la no pueden hacerlo solas y que necesitan orientaciones. Al preguntar tarea". (C24).	

Fase 2: en la fase educativa se desarrollaron siete talleres pedagógicos con agentes educativas y dos con acudientes. Las características sociodemográficas de los/las participantes se muestran en la Tabla 4. Los hogares comunitarios y Centros de Desarrollo Infantil están ubicados en barrios estrato bajo- bajo y bajo.

**Tabla 4:** Características sociodemográficas de los/as participantes.

Agentes educativas (n=25)		
Edad	Años de experiencia en ICBF	Nivel educativo
Mínima 31 años	Mínima 7 años	Profesionales 40% (n=10)
Máxima 65 años	Máxima 31 años	Técnicas 40% (n=10)
Entre 31 y 40 años 40% (n=10)	< 10 años 12% (n=3) Entre 11 y 20 48% (n=12)	Bachilleres 20% (n=5)
Entre 41 y 50 años 24% (n=6)	> 20 40% (n=10)	
Entre 51 y 60 años 24% (n=6)		
>60 años 12% (n=3)		
Cuidadores (n=55)		
Rol	Edad promedio	Nivel educativo
Madres de familia 78.18%(n=43)	28 años	Primaria 20% (n=11)
Abuelas 20.00%(n=11)	42 años	Primaria 64% (n=35) Bachillerato 36%(n=20)
Padre de familia 1.81% (n=1)	35 años	Bachiller 100(n=1)

Estas características fueron importantes para la trasposición didáctica en los talleres pedagógicos, el lenguaje utilizado y para la interpretación de sus narrativas.

Para el diseño de los talleres se plantearon preguntas orientadoras para la reflexión de acuerdo con el MCS y se utilizaron mensajes clave para estimular acciones preventivas. También, se tuvieron en cuenta los resultados de la fase 1, en la cual las participantes expresaron inquietudes y sugerencias de temas a abordar y metodologías a usar. Cada taller tuvo una duración de aproximadamente dos horas. El contenido de los talleres pedagógicos se resume en la Tabla 5.

**Tabla 5:** Talleres pedagógicos con agentes educativas y acudientes

Tema	Metodología Unidades de sentido. Talleres con agentes educativas.
Sesión 1.	Se realizó exposición con diapositivas por "A nosotros nos puso a formar la boca parte de los investigadores y conversatorio con los dientes, con la lengua y uno dice
Salud bucal integral, salud como derecho, anatomía del sistema estomatognático	con las participantes. Se elaboró una “eso tan fácil”, nada, si usted va y mira, boca en foamy del tamaño de un pliego no le cuadra (risas), pero de verdad es con partes desarmables, de manera que muy divertido y es muy entretenido". las agentes colocaban parte por parte y (AE2) explicaban su funcionamiento, ante lo cual los investigadores complementaban.
Sesión 2. Crecimiento y desarrollo: formación de los dientes desde la etapa prenatal, erupción dental, cambio de dentición, cuidados, consecuencia de fármacos. Lactancia materna y su relación con la salud bucal.	Se solicitó a las participantes escribir en un "La lactancia es exclusiva hasta los 6 papel una frase en la que compartieran sus meses, hasta los 2 años con alimentación conocimientos y experiencias sobre la lactancia complementaria. La leche materna contiene materna. Luego se hizo una plenaria. Se usaron el agua que nos niños necesitan. Posee las imágenes, radiografías y un video para mostrar primas vacunas. Es el principal vínculo el proceso de formación de los dientes. Se llevó a afectivo con la madre". (AE10) cabo la lúdica "mitos y realidades", que consistió "No sabíamos que la lactancia tenía relación en que las participantes debían escoger si pararse con el desarrollo maxilar". (AE12) a la derecha o a la izquierda de una línea trazada en el piso, un lado correspondía a mitos y el otro a realidades, según la postura que asumieran ante afirmaciones que se les daban. Después se hizo retroalimentación.
Sesión 3.	El tema fue ilustrado con numerosas fotografías. “Llevo muchos años trabajando con niños Se desarrolló una lúdica para la cual el grupo de 4 y 5 años y todavía toman tetero. No
Hábitos bucales deformantes: tetero, chupos, succión digital, respiración oral, onicofagia, relación con el crecimiento y desarrollo, relación con maloclusiones. Lavado de manos.	se dividió en dos equipos. Cada uno tenía una los cepillan, uno sabe. Tres madres me han cartelera en la pared y una serie de imágenes dicho: que se me va a desnutrir el niño, que recortadas. Cada equipo debía ubicar en la por su culpa no toma leche, que usted quién cartelera los hábitos bucales deformantes y al se cree, como se ve él de lindo con el tetero". frente las ilustraciones de sus consecuencias. (AE14) Posteriormente tuvo lugar una plenaria. Para el lavado de manos se hizo el proceso práctico de la técnica con el uso de gel antibacterial.
Sesión 4.	Se utilizó la herramienta virtual Kahoot, en la cual “Tan chévere esa actividad, ¿por qué no se diseñó un concurso con preguntas, cada una hicieron más preguntas?”. (AE6)
Repaso de los temas. Primera parte de la explicación de la formación de la caries con énfasis en la caries de infancia temprana. Formación de la placa bacteriana.	con cuatro opciones de respuesta. Estas fueron proyectadas en la pantalla con la que contaba el salón de reuniones. Por equipos las participantes respondieron desde sus celulares (esta actividad requería conexión a Internet). Luego, se explicó el proceso de desarrollo de la caries a través de imágenes.

### Talleres con acudientes

Se constató que las agentes educativas tenían amplios conocimientos acerca de la lactancia materna, sin embargo, desconocían su relación con la salud bucal. De igual manera, los acudientes tampoco tenían conocimiento al respecto. Por otro lado, el uso de imágenes de las fases de la caries, de las bacterias vistas a través de un microscopio y de la caries de biberón, generó impacto en ambos grupos de participantes. Los acudientes desconocían las graves consecuencias de que los niños/as se duerman con el tetero y sin cepillarse. Las imágenes acerca de las consecuencias de los hábitos bucales deformantes también llamaron la atención y facilitaron el diálogo a partir de las experiencias con sus hijos/nietos.

Por otro lado, con dinero del proyecto de investigación se adquirieron materiales didácticos que fueron entregados a las agentes educativas para que en cada uno de los hogares comunitarios ellas enseñaran a los niños/as prácticas de higiene bucal.

Fase 3: grupos focales con agentes educativas (ver Tabla 6).

**Tabla 6:** Fase 3, unidades de sentido identificadas en los grupos focales con agentes educativas.

Categorías	Análisis de las categorías
Creencias en Salud Bucal	“Fue un proceso que nos llevó a reflexionar, no solo a ir a escuchar y “ay es que vamos a hablar y hablar”, porque cuando empiezan a hablar y hablar uno llega un momento en que se desconecta de lo que están diciendo y uno no pone cuidado a los que explican. Como era tan interactivo lo que estábamos haciendo, que todos participábamos. Y cada vez que llegaban era con una práctica diferente, entonces fue bastante interesante”. (AE21). “Yo caí en cuenta de que no tenía argumentos, o sea, no tenía cómo sustentarle a un papá por qué debía cepillar al niño, pero como ya tengo argumentos, me funciona”. (AE14). “Aprendimos mucho, la importancia de que el niño se pegue del pecho, de la leche materna, lo importante que es también para sus dientecitos y para que fortalezca las encías. Eso fue algo que a mí se me quedó que era muy importante *(...) y* que desde que el niño nace hay que tener en cuenta limpiar las encías”. (AE20). “Uno sí coge más consciencia, tocamos temas muy a fondo, sobre las enfermedades que dan, sobre los cuidados de los dientes, entonces aprendimos muchas cosas que en realidad le cambia a uno el panorama, no para el trabajo, sino también para uno”. (AE11)
Susceptibilidad Percibida	“Hay niños que no tienen buena pronunciación, también porque no hicieron la lactancia materna, que el hecho de que los niños se amamanten hace que ellos desarrollen mejor todos los músculos y las partes que tienen que ver con la dentadura”. (AE4)
Severidad Percibida	“La niña que no tiene los dientecitos de adelante y ella mantenía muy acomplejadita y ni se reía porque los otros niños se burlaban de ella que porque estaba mueca”. (AE12). “Cuando un niño tiene los dientecitos malitos eso causa mucho dolor, eso causa malestar, eso causa que él no pueda disfrutar de las actividades, no puede disfrutar los alimentos”. (AE2)
Barreras Percibidas	“Yo tengo una mamá que con esa no pude, que no le da pesar no llevar al niño al odontólogo y tiene los dientes muy malitos, prefería retirarlo [del hogar comunitario] para que uno deje de molestarlos”. (AE9) “Me siento decepcionada porque yo cuando eso tenía un bebé que pasa con el dedo en la boca y no fui capaz con la mamá “no profe, yo también chupé dedo hasta vieja y no se me dañaron los dientes””. (AE7)
Beneficios Percibidos	“Niños felices, que si ellos tienen una boca sana tienen una autoestima”. (AE9)
Autoeficacia	“Ella me llevaba el carné, yo me ponía feliz, que yo creo que esa niña no había tenido cita jamás”. (AE14) “Ellos [los niños y niñas] muchas veces hacen mucho más caso que la mamá y cuando van a la casa dicen “es que la profe me enseñó así” entonces eso está repercutiendo en algo”. (AE22)

### Creencias en Salud Bucal

Algunas de las agentes todavía consideran que la salud bucal se limita a los hábitos de higiene, pero otras aprendieron que esta se relaciona con la salud general y el bienestar; asimismo, reconocieron que la salud bucal es un derecho. Igualmente, afirmaron que lo importante es tener rutinas en las familias y procurar que donde no hay acudientes atentos sean los infantes quienes soliciten el cepillado a los adultos.

En relación con las estrategias educativas desarrolladas, expresaron sentirse satisfechas con la metodología lúdica y los temas abordados. Mencionaron asombro con las imágenes y audiovisuales alusivos a los temas y expresaron que este proceso participativo y de reflexión fue útil para sus vidas. Las agentes dijeron sentirse más capacitadas para enseñar las patologías bucales, sus consecuencias y el por qué es necesaria la higiene bucal. Destacaron que anteriormente recomendaban el cepillado, pero que ahora enseñan la técnica adecuada.

Respecto a los cambios percibidos afirman que fue determinante invitar a los talleres a los acudientes que no eran receptivos con las recomendaciones porque se convirtieron en replicadores en los posteriores encuentros de la escuela de padres y "tomaron conciencia" al llevar a sus hijos/as a cita odontológica, cepillarlos antes de acostarlos y abandonar el hábito del tetero. Además, las agentes lograron que algunos niños/as perdieran el miedo de asistir a consulta odontológica. 

### Susceptibilidad Percibida

Las agentes aprendieron a reconocer la caries en sus primeras etapas, lo que les permite dialogar con los acudientes de forma oportuna. Además, afirman que adquirieron conocimientos de gingivitis, aftas, herpes labial, sarro y “apretamiento dental”, y que les pareció muy importante conocer las consecuencias de los hábitos parafuncionales. También, reforzaron el tema de la prevención de accidentes, para evitar traumas dentales.

### Severidad Percibida

En sus palabras describen la progresión de la caries y su severidad. Asocian los problemas de alimentación con la caries y argumentan sus consecuencias en los dientes permanentes. Tienen claro que la caries y los traumas dentales afectan no solo la salud física de los niños/as, sino también su bienestar emocional.

### Barreras Percibidas

Las agentes expresan que algunos acudientes amenazan a los niños/as con llevarlos al odontólogo o “arrancarles” un diente y que otros desconocen la importancia de la higiene bucal. Identificaron que los bajos ingresos económicos dificultan el cambio de cepillo o comprar seda dental. También, refieren como barrera aquellos odontólogos que debido a su trato no humanizado generan miedo en los niños/as.

### Beneficios Percibidos

Las agentes reconocen que los niños/as al tener su boca sana tienen beneficios como alimentación adecuada, mejora del autoestima y sociabilidad, adecuada fonación y, salud física y mental.

### Autoeficacia

Para ellas los primeros responsables del cuidado bucal de los infantes son las familias, pero también asumen su parte como educadoras, además, en sus discursos mencionan el sistema de salud y el Estado como garantes del derecho a la salud bucal, e involucran a toda la sociedad en la corresponsabilidad del cuidado. Por otra parte, consideran que los juguetes didácticos suministrados en el marco del proyecto sirvieron para despertar mayor interés y motivación en los niños/as.

Las agentes lograron que casi todos los acudientes llevaran a sus hijos/as a cita odontológica durante la ejecución de este proyecto. También, que los acudientes comprendieran que amenazarlos con consultas odontológicas era incorrecto. Varias compartieron sus logros con respecto a que algunos niños/as abandonaron el biberón y la succión digital.

## Discusión

El MCS ha sido utilizado para explicar por qué las personas se implican o no en acciones relacionadas con la salud^26^. En la presente intervención de educación para la salud se buscó con dicho modelo la incidencia en aspectos que podrían conllevar a cambios de percepciones, que en últimas conducirían a acciones saludables o actitudes de disposición a ellas. Por ejemplo, con la información brindada a los acudientes acerca de la severidad de la caries de biberón, estos en su mayoría concluyeron que es necesario cambiar el hábito de acostar a sus hijos/nietos con el tetero. De esta manera, los acudientes cambiaron la estimación de la probabilidad de que una acción (evitar el tetero en la noche y cepillar a sus hijos/nietos antes de dormir) los conduzca a una meta (evitar la caries de infancia temprana). El MCS se puede traducir de la siguiente manera: “a) el deseo de evitar la enfermedad b) la creencia de que una conducta saludable específica puede prevenir la enfermedad (o si se está enfermo, la creencia de que una conducta específica puede aumentar la probabilidad de recuperar la salud)”^26^.

En cuanto a las dimensiones del modelo puede afirmarse que antes de la intervención educativa las agentes, según la susceptibilidad percibida, estimaban que los niños/as enfermaban no solo de caries sino también de aftas, gingivitis, amigdalitis, herpes y fluorosis, además reconocían la vulnerabilidad a los traumas. Igualmente, identificaban el tetero y el chupo como hábitos nocivos para la salud bucal. Con la intervención educativa profundizaron conocimientos en estos aspectos, el por qué y cómo se desarrollan dichas patologías, cómo reaccionar ante accidentes en el hogar comunitario y las consecuencias de los hábitos bucales deformantes.

Por otro lado, los acudientes dieron cuenta de patologías como la caries y los cálculos, pero no consideraban que sus hijos/nietos las sufrían, es decir, la percepción de susceptibilidad era baja antes de participar en los talleres pedagógicos. Con respecto a la severidad, las agentes mencionaron que la caries genera en los niños/as dolores, imposibilidad de masticar, burlas de los compañeros, miedo al odontólogo, afectación de la autoestima y de la socialización. En igual sentido, los acudientes advirtieron presencia de dolor, ardor y problemas para la alimentación. Sin embargo, sobre las demás patologías o hábitos bucales no había conocimiento acerca de la gravedad, lo cual se reforzó con las actividades educativas. Conocer estos aspectos es relevante dado que para el desarrollo de comportamientos saludables en la niñez es fundamental la consolidación de escenarios familiares y comunitarios en los que se promuevan conductas salutogénicas, toda vez que se trata de un proceso en el que los niños/as son influenciados por su contexto^27^.

Por otra parte, las barreras percibidas por las agentes se resumen en dificultades logísticas en el hogar comunitario, falta de acompañamiento de los acudientes para el cuidado bucal de los niños/as y miedo de los infantes al odontólogo/a. Mientras que los acudientes señalaron la falta de atención humanizada en la consulta odontológica, desgano de algunos niños/as en cepillarse y dificultad de cepillar la lengua. En el transcurso de los talleres estas barreras fueron abordadas para generar claves de acción que permitieran superarlas; sin embargo, persistió la dificultad para usar la seda dental y el desinterés de algunos padres/madres de familia por la salud bucal de sus hijos/as. Los acudientes solo identificaron la posibilidad de evitar dolor y asistencia a la consulta. Finalmente, acerca de la autoeficacia las educadoras se sienten capaces de coadyuvar a la salud bucal de los niños/ as, no obstante, creen que requieren mayor cooperación de los acudientes. Al finalizar la estrategia educativa se pudo constatar que la percepción de autoeficacia mejoró tanto en las agentes como en los acudientes y que ambos realizaron acciones de forma más articulada. Al respecto, Ávila et al.^28^ mencionan que la adquisición de hábitos saludables en la niñez requiere de trabajo en casa y articulación entre la escuela y la comunidad, de allí que recomienden la realización de estrategias para mejorar la autoeficacia de los cuidadores.

Si bien la literatura científica muestra que el MCS es efectivo para mejorar la salud bucal, las revisiones sistemáticas y meta-análisis basadas en estudios cuantitativos no profundizan acerca del enfoque pedagógico utilizado en las investigaciones^21^^,^^22^^,^^29^. En este sentido, cabe destacar que este proyecto se estructuró con metodología participativa basada en la comunidad a través de elementos lúdicos, como juegos serios (educativos) adaptados a los adultos para abordar diversos temas en salud bucal de la primera infancia. Esto se aleja de aquella pedagogía tradicional que Freire llamó “bancaria”, la misma que considera que las comunidades son objetos de intervención, sujetos pasivos a los cuales se les brinda información para que sea memorizada, y está basada en una relación vertical entre investigador (educador) y comunidad (educandos), en la que el primero es portador de verdad gracias al conocimiento científico^30^. Por lo contrario, en la educación popular se estipula que toda persona posee saberes derivados del mundo que habita y de su experiencia diaria. Por ende, en este tipo de proyectos educativos se fomenta el diálogo de saberes y el compartir de experiencias.

La orientación pedagógica planteada en este proyecto se acerca a la educación popular, que pretende forjar pensamiento propio y comprensión de las realidades próximas, en lugar de copiar o imitar teorías generadas por fuera de los contextos latinoamericanos^31^. Por ello, contemplar la experiencia y las vivencias en la investigación significa considerarla como pedagogía, más allá de reducir los aprendizajes a cifras. En otras palabras, se trata de cómo los sujetos involucrados resignifican sus prácticas y su cotidianidad con los aprendizajes, tal como lo narraron las agentes educativas partícipes en este proyecto, quienes incorporaron nuevos saberes a sus prácticas como maestras, como orientadoras en las comunidades, como madres y como ciudadanas para incidir en la promoción de la salud en la primera infancia. En este sentido, “se afirma que no existe acción educativa y pedagógica sin contexto”^32^.

No obstante, hay que reconocer como limitante que la presente investigación no llega a instancias de emancipación y liberación, desde la óptica de la pedagogía popular crítica, como tampoco a nuevas configuraciones de subjetividades o de formas organizativas para enfrentar al poder hegemónico. Sin embargo, no por ello se pueden desconocer las transformaciones generadas en el contexto de los sujetos participantes, no solo en la adquisición de nuevos conocimientos, sino también en cambios de prácticas, de formas de relacionarse (ej. con los profesionales de la salud), en la adquisición de argumentos de la salud bucal como derecho y en la comprensión de la corresponsabilidad de todos los miembros de la sociedad y del Estado frente a la salud de los niños y niñas.

Asimismo, puede afirmarse que en este proyecto se desmitificó el saber científico para que estuviera al alcance de comunidades no académicas, para que pudieran utilizarlo en su cotidianidad y para la resolución de problemas. Por ello, en este estudio la metodología usada tiene pertinencia estratégica y potencial transformador. En proyectos participativos como el presente, la reflexión acerca de la metodología es fundamental en función de los objetivos, la cultura, los participantes y el referente teórico escogido. Por lo tanto, la organización estratégica de las acciones es primordial en el método de IA y en la educación popular, estas dos fueron las orientaciones ideológico-políticas para el proyecto de investigación, porque “tan importante como aquello que se quiera lograr, es lo que se hace para lograrlo”^32^.

Esta investigación debe entenderse como un modelo de intervención que demostró cómo se puede ajustar una estrategia educativa a las necesidades de la comunidad y cómo esto facilita la participación y la aceptabilidad. Como fortaleza cabe mencionar que hasta el momento existen pocas investigaciones en Latinoamérica que tienen como referente el MCS, menos aún que aborden temas de salud bucal y primera infancia^33^. Sin embargo, este modelo tiene una limitación por cuanto concentra las responsabilidades para mejorar la salud en los individuos.

## Conclusiones

La salud bucal de los niños y niñas está supeditada a las acciones que realicen los cuidadores y maestras. Las creencias en salud bucal recopiladas demuestran conocimientos previamente adquiridos, aunque con algunos vacíos en cuanto a prácticas de higiene bucal, causas de las enfermedades bucales, tratamientos y consecuencias. Los participantes, casi en su totalidad mujeres, demostraron interés en vincularse activamente en los talleres pedagógicos diseñados con estrategias lúdicas para fomentar el “aprender haciendo”. Tanto agentes educativas como acudientes refirieron la aplicabilidad de los nuevos saberes en el cuidado bucal de los infantes a través del cambio en prácticas cotidianas como el cepillado dental y la disminución del biberón.

En tal efecto, se recomienda el diseño de estrategias educativas que, guiadas por modelos cuya efectividad ha sido comprobada en la literatura científica, utilicen metodologías participativas, adaptadas a las necesidades y contextos de las comunidades, pues se requiere producir conocimiento situado y con aceptabilidad cultural.
